# A *Dioscorea opposita* Thunb Polysaccharide-Based Dual-Responsive Hydrogel for Insulin Controlled Release

**DOI:** 10.3390/ijms23169081

**Published:** 2022-08-13

**Authors:** Wei Liu, Xiaoge Wang, Danyang Zhou, Xiangze Fan, Jinhua Zhu, Xiuhua Liu

**Affiliations:** Henan International Joint Laboratory of Medicinal Plants Utilization, College of Chemistry and Chemical Engineering, Henan University, Kaifeng 475004, China

**Keywords:** *Dioscorea opposita* Thunb polysaccharide, hydrogel, insulin, drug delivery

## Abstract

A novel hydrogel (DOP/PEI-PBA) based on the “three-component” reaction of 2-formylphenylboric acid (2-FPBA), the primary amine group of polyethyleneimine (PEI) and the cis-o-dihydroxy groups of *Dioscorea opposita* Thunb polysaccharide (DOP) was designed in this work. The hydrogel can be easily prepared by simply mixing the three reactants at room temperature. The hydrogel had dual responsiveness to glucose and pH, and can realize the controllable release of insulin. Moreover, the hydrogel combining insulin and DOP can inhibit the reactive oxygen species (ROS) level and malondialdehyde (MDA) content, and promote glucose consumption as well as the level of superoxide dismutase (SOD), in high-glucose-induced injury in HL-7702 cells, which reflects the synergistic effect of insulin and DOP to protect hepatocytes from oxidative stress at the same time. Further in vitro cytotoxicity studies showed that the hydrogel had good biocompatibility and no obvious toxicity to cells. These indicate that the prepared hydrogel (DOP/PEI-PBA) can be expected to be applied in the clinical treatment of insulin deficiency in diabetes.

## 1. Introduction

Diabetes is the third most prevalent global endemic disease after cancer and cardiovascular disease, and is mainly characterized by the dysregulation of blood glucose and blood lipid metabolism. In recent years, the number of patients with diabetes has increased rapidly, with 4.2 million deaths from diabetes in 2019 [1]. At present, there is still no cure for diabetes; subcutaneous injection of insulin remains the main method to control insulin-dependent diabetes in the clinic [2]. However, the poor liposolubility, inactivation and high molecular weight of injected insulin reduce its bioavailability, leading to chronic complications in diabetes [3]. Meanwhile, this means of administration may bring pain and infection at the injection site, which can easily cause psychological tension in patients with diabetes [4]. Moreover, it is also important to precisely control the administration dosage of insulin, as high or low doses may easily cause hypoglycemia or hyperglycemia [5]. On the other hand, as a type of protein drug, insulin can be easily degraded and inactivated by various digestive enzymes in the gastrointestinal tract with oral administration [6]. Moreover, due to the relatively large molecular weight of insulin, it has poor permeability to intestinal epithelial cells and is difficult to be absorbed [7]. Thereby, oral insulin delivery systems are not effective in clinical application. Therefore, it is of great significance to develop a new type of insulin delivery system to reduce the injection frequency and realize the controlled release of insulin according to the rapid change in blood glucose.

The glucose-responsive systems have been widely investigated in the field of insulin delivery, as they can control drug release with the fluctuations in blood glucose. Materials containing phenylboronic acid (PBA) have been extensively used as glucose-responsive materials for insulin delivery due to their ability to form dynamic borates with cis-diol compounds [8,9,10]. Kuivila et al. first reported that PBA can be specifically reacted with diols to form a phenylborate complex [11], which has the advantage of a reversible glucose response for self-regulated controlled drug release. In the past few years, the functional PBA groups have usually been incorporated and connected to the polymer crosslinking network to endow the system with glucose-sensitive properties for insulin delivery. Kataoka et al. used polypropylene to prepare a glucose-sensitive carrier based on PBA, and successfully realized the “on–off” control of insulin release [12]. Lu et al. copolymerized dimethylaminoethyl methacrylate (DMAEMA) with 3-acrylamidephenylboronic acid (AAPBA) to prepare temperature-, pH- and glucose-sensitive hydrogels, and it was concluded that the gel showed good glucose sensitivity under physiological conditions [13]. Additionally, a glucose-responsive hydrogel (*p* (APBA-b-LAMA)) was synthesized by copolymerization of 3-acrylamidophenyl boronic acid with 2-lactobionamidoethyl methacrylate, and the hydrogel displayed glucose-dependent insulin release [14]. However, the poor biodegradability of polyacrylic acid materials limited its clinical application [15]. Good biocompatibility is necessary for the application of glucose-sensitive drug carrier materials in insulin delivery systems. Polysaccharides, such as cellulose and its derivatives [16,17], lignin [18], pectin and starch [19], have been selected as some of the most suitable materials for the construction of hydrogels, mainly due to their abundance, low cost, tailorability and biocompatibility [20].

*Dioscorea opposita* Thunb polysaccharide (DOP) is one of the main active components in *Dioscorea opposita* Thunb, which is a traditional herbal medicine [21]. There are monosaccharides such as β-1,3-glucose, α-1-galactose and α-1,6-galactose in purified DOP [22], which contains abundant cis-diol groups. In addition, subsequent reports indicated that DOP had a modulating effect on the improvement in glucose metabolism and insulin sensitivity in rats receiving a high-fructose diet by reducing inflammatory protein products (including levels of IL-1β, TNF-α) and increasing the levels of antioxidant enzyme (SOD) activity [23,24,25]. Moreover, DOP was also expected to serve as a natural thickener and emulsifier in the food industry due to its good “gel-like” behavior and emulsification properties [26,27].

In this paper, a hydrogel based on the “three-component” reaction of 2-formylphenylboric acid (2-FPBA), the primary amine group and cis-o-dihydroxy groups was prepared. The DOP/PEI-PBA hydrogel is proposed as a delivery vehicle for insulin for sustained release. This hydrogel was formed by a 3D network structure based on dynamic borate ester bonds. Both a high concentration of glucose and low pH can break the borate ester bonds and destroy the network structure, and then the loaded drug can be continuously released with a glucose and pH response. Furthermore, the synergistic effect of polysaccharide and released insulin after boronate bond cleavage could suppress the ROS level and MDA content, and promote glucose consumption and SOD levels, following high-glucose-induced injury in HL-7702 cells.

## 2. Results and Discussion

### 2.1. Synthesis and Characterization of Hydrogels

**Figure 1:**
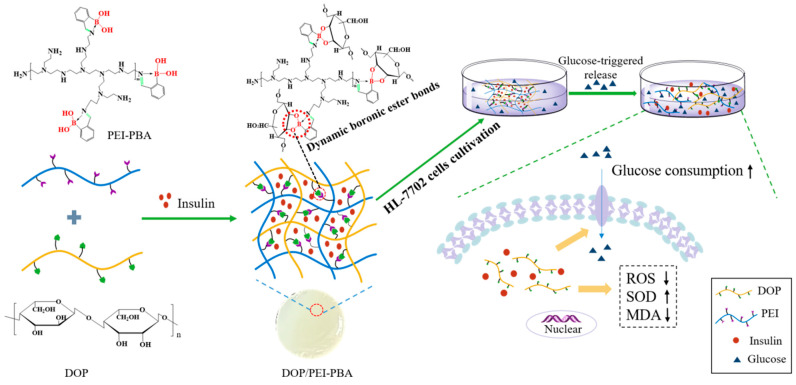
Schematic illustration of DOP/PEI-PBA hydrogel for delivery of insulin to protect against high-glucose-induced injury in HL-7702 cells.

As displayed in Figure 1, 2-FPBA was firstly reacted with the primary amine group of PEI to obtain PEI-2-FPBA. Subsequently, the boric acid groups were reacted with cis-diol groups on the DOP to form boric acid ester bonds. Finally, the hydrogel was formed by imide and boronate ester bonds. In order to investigate the properties of the hydrogel, hydrogels with different proportions of PEI and PBA were prepared.

**Figure 2:**
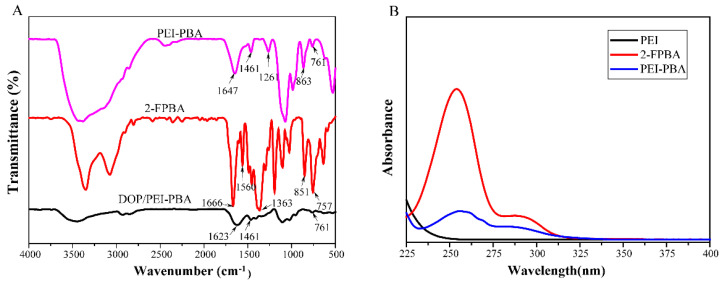
FT-IR spectra of 2−FPBA, PEI-PBA and DOP/PEI−PBA hydrogel (**A**) and UV–vis spectra of PEI, 2−FPBA and PEI−PBA (**B**).

The FT-IR spectra of 2-FPBA, PEI-PBA and DOP/PEI-PBA are presented in Figure 2A. For 2-FPBA, the peak at 1666 cm^−1^ was attributed to C=O stretching vibration [28]. The absorption peak at 1363 cm^−1^ was assigned to –B(OH)_2_ stretching vibrations, and the absorption bands at 851 cm^−1^ and 757 cm^−1^ were aromatic C-H out-of-plane bending bands, while 1560 cm^−1^ was the characteristic absorption band of the benzene ring [29]. In addition, for PEI-PBA, the peak at 1461 cm^−1^ was the characteristic absorption peak of the B–O bond and 1261 cm^−1^ belonged to the B–O–H bond [30]. The stretching vibrations of C=N were located at 1647 cm^−1^. In addition, the deformation vibration peaks of the benzene ring proton were also displayed at 863 cm^−1^ and 761 cm^−1^, respectively. Moreover, for DOP/PEI-PBA, the characteristic peaks at 1623 cm^−1^ and 1461 cm^−1^ were ascribed to the C=N stretching vibration and B–O–C asymmetric stretching vibration, respectively, proving that imine and borate moieties were formed in the polymer network [31]. All these results showed that the grafting and crosslinking reaction took place between DOP and PEI-PBA, indicating that the hydrogel was successfully prepared.

The UV–vis absorption spectrum of the PEI-PBA is demonstrated in Figure 2B. It shows that the benzene ring had an absorption peak in the range of 230~270 nm. The PEI-PBA polymer displayed an absorption peak similar to 2-FPBA at 255 nm, while PEI did not have any absorption at this wavelength, indicating that PEI and PBA reacted, and PEI-PBA polymers with phenylboronic acid groups were successfully prepared.

**Figure 3:**
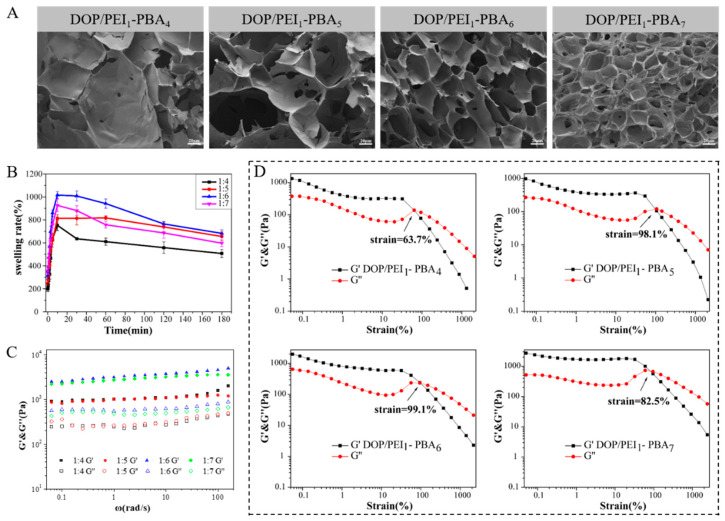
Characterization of DOP/PEI-PBA hydrogels: SEM images (**A**), swelling ratios (**B**), rheological analysis of DOP/PEI-PBA hydrogel in frequency sweep mode (**C**) and strain sweep mode (**D**) of hydrogels with different ratios of PEI to PBA.

In order to further characterize the morphology of the hydrogels, SEM images of the freeze-dried hydrogels were obtained, as displayed in Figure 3A. As shown in the images, a honeycomb porous microstructure could be observed in the DOP/PEI-PBA hydrogels due to the crosslinking effect of the borate ester bond and the effect of water molecules. Moreover, with the increase in the amount of crosslinking agent, the porous structure became denser and the pore size decreased. It is well known that a 3D architecture with highly interconnected pores provides sufficient internal space for the controlled release of protein drugs, and can be widely used in cell engineering [32].

### 2.2. Swelling and Rheological Behavior of DOP/PEI-PBA Hydrogels

Generally speaking, the crosslinking density of the gel network will affect the properties of the hydrogel, including swelling and rheological behavior [33,34]. In this study, 2-FPBA, as the crosslinking agent of the DOP/PEI-PBA hydrogel, was mainly investigated with different consumption levels.

The swelling of hydrogels with different crosslinking densities was detected. As shown in Figure 3B, the hydrogels reached the swelling equilibrium in a relatively short time, and the swelling rate increased with the increase in the amount of crosslinking agent 2-FPBA, and reached the maximum when the molar ratio of PEI to PBA was 1:6. However, when the ratio of PEI to PBA was 1:7, the increase in crosslinking points led to a tight network structure in the hydrogel, which was not conducive to swelling, and the swelling rate decreased instead.

Generally, dynamic measurements such as the elastic and viscous properties of materials are often used to describe the rheological behavior. The elastic modulus (G’) and viscous modulus (G″) of hydrogels were tested using a rheometer to reflect the mechanical strength of the hydrogel with different degrees of PBA substitution. As shown in Figure 3C, for all hydrogels, the G’ values were significantly greater than the G″ values, indicating that stable hydrogels had been successfully formed [26]. The elastic modulus (G’) of a hydrogel is mainly affected by the number of crosslinking points. Within a certain degree, the value of G’ increases with the increase in crosslinking density. The G’ value of DOP/PEI_1_-PBA_6_ was the largest. However, when the amount of PBA was further increased, the G’ value of the hydrogel (DOP/PEI_1_-PBA_7_) decreased. It may be that the free phenylboronic acid affected the elasticity of the hydrogels due to the high content of PBA. The larger the value of G’, the greater the elasticity of the material [35].

Figure 3D shows the shear strain behavior of the hydrogel at strain amplitude sweeps. The decrease in the G’ value and the increase in the G″ value near the intersection of G’ and G″ indicated the collapse of the hydrogel structure, and the hydrogel changed from a solid with gel characteristics to a liquid-like substance with fluid characteristics [36]. The critical strain of the DOP/PEI_1_-PBA_6_ hydrogel was the maximum (99.1%), while the DOP/PEI_1_-PBA_4_ hydrogel collapsed at a strain of 63.7%. The larger the critical strain, the greater the deformation of the hydrogel due to the shear stress, which means that the DOP/PEI_1_-PBA_6_ hydrogel can withstand greater deformation. Considering its better elastic modulus, the higher critical strain and better swelling rate, DOP/PEI_1_-PBA_6_ hydrogel was selected for the following applications.

According to the reference [37], excessively high or low water content in a hydrogel is not conducive to its swelling equilibrium. When the water content is too high, the hydrogel almost expands completely, resulting in the relatively low toughness of the hydrogel, such as in the DOP/PEI_1_-PBA_4_ hydrogel. Meanwhile, when the density of the crosslinking agent is higher, the water content is lower, the hydrogel will shrink, and thus the toughness will be reduced.

### 2.3. Degradation of Hydrogels

**Figure 4:**
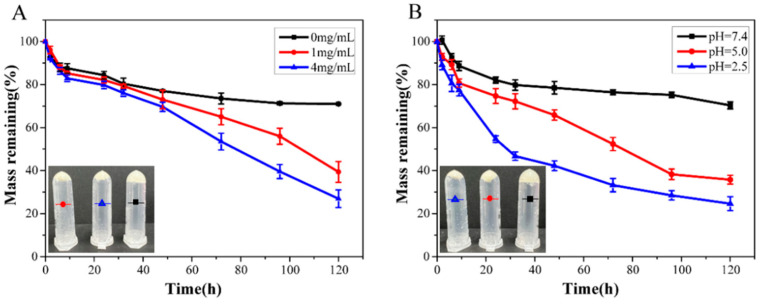
The degradation of hydrogels at different glucose levels (**A**) and pH (**B**).

It was reported that the phenylborate ester bond is a type of dynamic chemical bond sensitive to pH, oxidation and glucose [38,39]. This means that DOP/PEI-PBA also has good multiple-response performance. The effects of glucose concentration and medium pH on the hydrogels were investigated. The degradation results are shown in Figure 4. It can be seen that the hydrogel could be degraded in pH 7.4 buffer solution. However, the degradation rate was slow. After 120 h, more than 70% of the hydrogels still remained. The degradation occurred mainly due to the hydrolysis of imines in PBS [40,41]. When the glucose concentration increased in the solution, the degradation rate of the hydrogel increased significantly. As the borate ester bond is a dynamic bond, with the increase in the glucose concentration, glucose molecules will compete with DOP to react with PBA molecules, so that DOP can be dissociated from the hydrogel, resulting in the degradation of the hydrogel. After 120 h in 4 mg/mL glucose solution, 76% of the hydrogel was degraded. It can also be clearly seen from the illustrations in Figure 4A that the amount of hydrogel decreased significantly after 24 h. The change in buffer pH had a more obvious effect on the degradation of the hydrogel. As demonstrated in Figure 4B, the degradation rate of the hydrogel increased significantly with the decrease in pH. In an acidic solution, phenylboric acid is transformed from SP^3^ hybrid to SP^2^ hybrid, which is a planar triangular structure, resulting in ring dissociation with the diol groups of DOP, so the hydrogel is degraded. In addition, 78% of the hydrogel was degraded in pH 2.5 solution after 120 h. The in vitro degradation results indicated that the DOP/PEI-PBA hydrogel has clear multiple-response properties and is expected to be applied as a carrier for controlled drug release.

### 2.4. In Vitro Release of Insulin from Hydrogel

**Figure 5:**
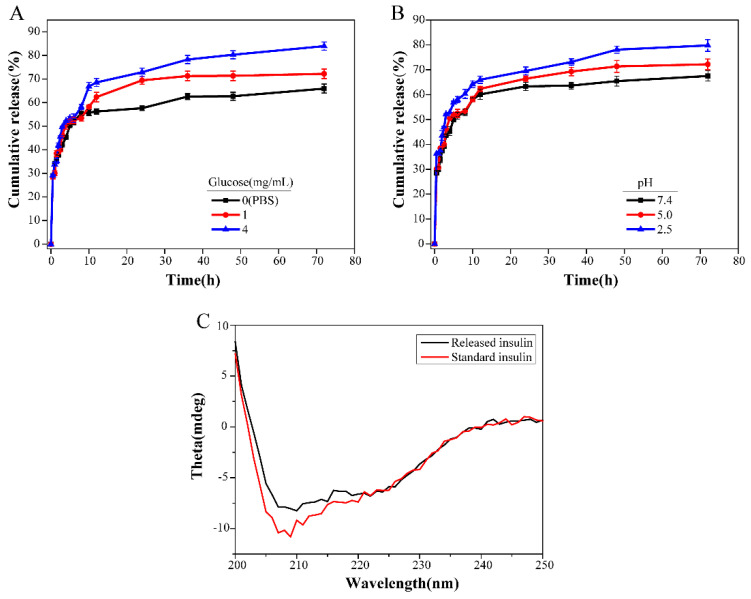
Cumulative release of insulin from the DOP/PEI−PBA hydrogel at different glucose levels (**A**) and pH (**B**). CD spectra of standard and released insulin (**C**).

The release profiles of insulin from hydrogels in different media are shown in Figure 5. It can be observed that in PBS medium without glucose at pH 7.4, 55.7 ± 1.13% insulin was initially released within 10 h, and then showed a sustained and slow release trend within 3 days due to the rapid diffusion of the drug near the hydrogel surface. In contrast, insulin was released much faster from hydrogels in the presence of a certain concentration of glucose and a low-pH solution. At a high concentration of glucose (4 mg/mL) and pH 2.5 PBS medium, the cumulative release of insulin reached 83.9 ± 1.71% and 79.8 ± 1.39%, respectively, within 72 h. These results indicated that glucose could easily diffuse into the hydrogel matrix and competitively combine with PBA to form borate ester bonds, destroying the 3D network structure of the hydrogel [9], so as to achieve the sustained release of insulin as needed in response to high blood glucose levels.

Insulin released in 24 h was collected, and the stability of insulin released from the hydrogels was tested by circular dichroism (CD) spectroscopy. As shown in Figure 5C, there was no significant difference in the secondary structure of released insulin compared with standard insulin. This indicated that insulin had conformational stability during loading and release.

The kinetics of insulin release from the hydrogels were evaluated with different models, including the Ritger–Peppas model (Equation (1)), Sigmoidal model (Equation (2)), zero-order model (Equation (3)) and first-order model (Equation (4)).

(1) $${Q=Kt}^{n}$$

(2) $$Q=\frac{R_{s}}{{1+e}^{-k_{s}(t-t_{50})}}$$

(3) $$Q=k_{0}t$$

(4) $$ln\left(1-Q\right)=k_{1}t$$

where *Q* represents the cumulative release percentage of time *t* (h), *K*, *k_s_*, *k*_0_ and *k*_1_ are the corresponding kinetic constants and *n* is the diffusion index. The theoretical maximum release rate is *R_S_* (%), and *t*_50_ is the release kinetic constant. 

As shown in Table 1, the release of insulin in hydrogels fitted well with the Sigmoidal model, as it was immediately released during the first 10 h before reaching a plateau. The Sigmoidal release function describes anomalous non-Fickian diffusion. This anomalous phenomenon is caused by the changes in the polymer structure, including swelling, drug diffusion and internal stress caused by structural changes. In anomalous non-Fickian diffusion, the time scales of macromolecule relaxation and diffusion are similar [42]. The swelling of the polymer matrix also depends on the release medium.

### 2.5. In Vitro Cytotoxicity Test

**Figure 6:**
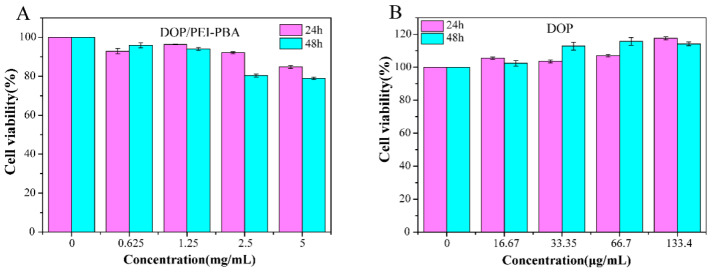
Cell viability of HL-7702 cells treated with DOP/PEI-PBA hydrogel extraction (**A**) and DOP (**B**) in different concentrations at 24 h and 48 h.

The biocompatibility of the hydrogel was evaluated by the MTT method. We investigated the cytocompatibility of the hydrogels with human liver cells (HL-7702) (Figure 6). Compared with the blank group, the cell survival rates of the DOP groups were all close to 100%, or even exceeded 100%, indicating that DOP can promote the proliferation of HL-7702 cells (Figure 6B). As expected, the cell viabilities of hydrogels with different concentrations were all higher than 80.0% within 48 h (Figure 6A), which confirmed the biological safety of the hydrogels.

### 2.6. The Protective Effect of DOP and Insulin on Hepatocytes

**Figure 7:**
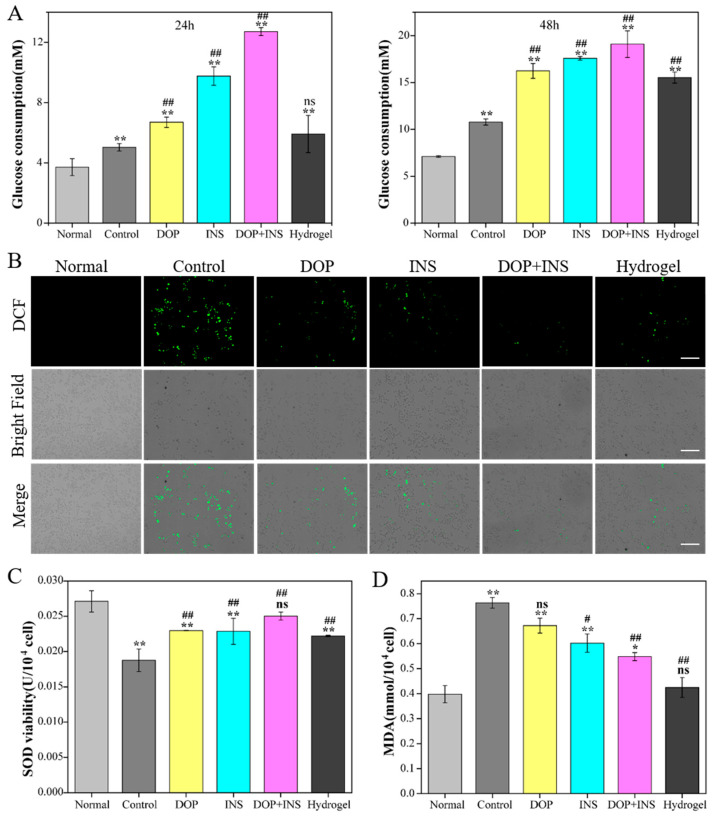
Effect of different formulations on glucose consumption (**A**), ROS (**B**), SOD viability levels (**C**) and MDA levels (**D**) in high-glucose-induced HL-7702 cells (* *p* < 0.05, ** *p* < 0.01, compared with normal group; # *p* < 0.05, ## *p* < 0.01, compared with control group; ns means no significance).

The glucose consumption rate indicates the ability of cells to uptake glucose [43]. Insulin can promote the consumption of intracellular glucose; however, it is difficult to maintain long-term high activity due to its short half-life [44]. As demonstrated above, in the presence of higher concentrations of glucose, the hydrogel will degrade and release the loaded insulin, and DOP will also be dissociated from the borate ester bond. Thus, the protective effect of DOP and insulin on HL-7702 cells treated with high glucose was investigated. As shown in Figure 7A, the longer the time period, the more glucose was consumed in each group. Compared with the control, both DOP and insulin could promote glucose consumption, and insulin was more effective. Furthermore, the glucose consumption of HL-7702 cells co-cultured with insulin and DOP increased significantly. In addition, after treatment with the hydrogel for 48 h, the glucose consumption rate was significantly increased due to the slow release of insulin compared with the control group. This fully demonstrated that the combined action of insulin and DOP made cells more active, thus promoting glucose consumption in cells.

Long-term exposure to a high-glucose environment would lead to the excessive production of reactive oxygen species (ROS) [45]. As shown in Figure 7B, obvious ROS generation was observed after 24 h of high-glucose treatment, and the green fluorescence intensity of each group decreased after treatment with DOP or insulin. It was observed that ROS levels decreased significantly when insulin and DOP were used together.

It is well known that the increase in ROS would lead to changes in the expression and activity of antioxidant enzymes superoxide dismutase (SOD) and malonaldehyde (MDA), which are indicators related to oxidative stress [46]. Figure 7C demonstrates the changes in SOD and MDA levels in HL-7702 cells. Compared with the control group, the intracellular SOD content increased significantly after treatment with different solutions, especially in the insulin and DOP combined group. Furthermore, there was no significant difference between the insulin and DOP combined group and the normal group. However, MDA levels were obviously reduced. These results indicated that the combination of insulin and DOP had a synergistic protective effect against HL-7702 cell injury induced by high glucose. 

## 3. Methods and Materials

### 3.1. Reagents and Apparatuses

Fresh *Dioscorea opposita* Thunb was purchased from Bao He Tang (Jiaozuo) Pharmaceutical Co. Ltd., Henan province, China, in November 2020. Insulin (porcine pancreas) was purchased from Shanghai Yuanye Bio-Technology Co., Ltd. (Shanghai, China). Polyethyleneimine and 2-formylbenzeneboronic acid were obtained from Aladdin Biochemical Technology Co., Ltd. (Shanghai, China). Dulbecco’s modified eagle medium (DMEM), 3-(4,5-Dimethyl-thiazol-2-yl)-2,5-diphenyl tetrazolium bromide (MTT), reactive oxygen species (ROS) assay kit, superoxide dismutase (SOD) and malondialdehyde (MDA) assay kit were all obtained from Beijing Solarbio Co., Ltd. (Beijing, China). Fetal bovine serum (FBS) was acquired from Tanhang Biotechnology Co., Ltd. (Zhejiang, China). Enzyme-linked immunosorbent assay (ELISA) kits were obtained from the Nanjing Jiancheng Bioengineering Institute (Nanjing, China). The human normal liver (HL-7702) (Procell CL-0111) cell lines were kindly provided by Procell Life Science & Technology Co., Ltd. (Wuhan, China). All other chemicals used were of analytical grade.

A Fourier transform infrared spectrometer (Bruker, VERTEX 70) was used to acquire the FT-IR spectra. A TU-1900 spectrophotometer (Beijing, China) was applied to obtain the UV–vis absorption spectra. A JSM-7610F (JEOL, Akishima, Japan) scanning electron microscope (SEM) was used to characterize the shapes of the hydrogels. The rheological properties of the DOP/PEI-PBA hydrogel were measured by a rotating rheometer (TA, DHR2) with a diameter of 25 mm at room temperature [47]. The frequency scanning was carried out in the range of 1–100 rad/s, and the strain was 0.1%. In addition, a strain amplitude sweep analysis was performed at a frequency of 1 rad/s in the strain range of 0.1 to 1000% to evaluate the mechanical strength of the hydrogel. The stained cells were imaged by an inverted fluorescence microscope (DMI8, Leica, Wetzlar, Germany). A circular dichrometer (AVIV, Model 420F, USA) was used to obtain the secondary structure of insulin.

### 3.2. Preparation of Dioscorea opposita Thunb Polysaccharide and DOP/PEI-PBA Hydrogel

Briefly, the polysaccharide was extracted with deionized water and precipitated with 60% ethanol according to the method previously described by Ma et al. [48]. Then, Sevag reagent and AB-8 resin were used to remove protein and pigment, respectively. Finally, the polysaccharide with a certain molecular weight was obtained by dialysis (8–14 kDa) for 7 days and stored for further use.

The DOP/PEI-PBA hydrogel was prepared as follows. Firstly, 150 mg/mL PEI and 15 mg/mL 2-FPBA were mixed in different molar ratios (1:4, 1:5, 1:6 and 1:7) and vortexed to obtain a uniform mixture, which was stored overnight at room temperature to allow a complete reaction. Finally, DOP/PEI-PBA hydrogels with different ratios of PEI and 2-FPBA were prepared by reacting PEI-PBA with DOP (4.0 wt %). The preparation of the insulin-loaded DOP/PEI-PBA hydrogel was essentially the same as the above process, except that insulin (1.0 mg) was added to the PEI-PBA mixture solution (1.0 mL) under mild stirring at 4 °C before adding the DOP solution (1.0 mL). 

### 3.3. Swelling Tests

A certain amount (Wd) of lyophilized hydrogel was immersed in a pH 7.4 buffer solution. The weight of the hydrogel at regular intervals (Wt) was recorded until the swelling equilibrium was reached. The swelling ratio of the hydrogel was calculated according to the following formula [49]:

Swelling ratio = (Wt − Wd)/Wd × 100(5)

where Wd is the weight of the dry hydrogel, and Wt refers to the weight of the swelling hydrogel at a certain time. 

### 3.4. Glucose- and pH-Mediated Hydrogel Degradation

Glucose with different concentrations (0 mg/mL, 1.0 mg/mL and 4.0 mg/mL, pH 7.4) and PBS solutions with different pH (2.5, 5.0, 7.4) were selected as the degradation media for hydrogels. The hydrogel (0.3 g) was placed in a centrifuge tube, and 2.5 mL of degradation solution was added to the tube with shaking at 37 °C; then, within a certain time interval, the degradation solution was removed completely and the hydrogel was weighted.

### 3.5. In Vitro Insulin Release from Hydrogels

First, 0.3 g hydrogel loaded with insulin (200 μg) was placed in 2.5 mL PBS solution (0.1 M, pH 7.4) with different concentrations of glucose (0 mg/mL, 1 mg/mL, 4 mg/mL) or 0.3 g hydrogel loaded with insulin was placed in 2.5 mL PBS solution (0.1 M) with different pH (2.5, 5.0, 7.4). The solution was shaken in the dark at 37 °C, and 250 μL aliquots were drawn at predetermined time intervals to test the amount of insulin released; the same volume of corresponding solution was replenished at the same time to keep the release medium volume constant. The content of released insulin was detected using an ELISA kit, according to the instructions. In addition, the secondary structure of the released insulin was tested with a circular dichrometer.

The cumulative release efficiency was calculated using the following equation [50]:

(6) $$\mathrm{Cumulative}\;\mathrm{release}\;(\%)=\frac{C_{n}\times V_{0}+V_{i}\sum_{i=1}^{n-1}C_{i}}{m}\times 100$$

where *C_n_* (µg/mL) represents the insulin sampling concentration at a specific time. *V*_0_ refers to the total volume of the release medium and *V_i_* (mL) is the sampling volume. Moreover, *m* (mg) represents the drug mass in the hydrogel.

### 3.6. In Vitro Cytotoxicity Test

The MTT assay was used to evaluate the cytotoxicity of the hydrogel. HL-7702 cells were seeded in 96-well microplates at a density of 3 × 10^3^ cells per well; after incubation for 24 h at 37 °C, the culture medium was replaced with 200 μL of culture medium containing different concentrations of PEI-PBA, DOP or hydrogel solution and coincubated for another 24 h and 48 h, respectively. Finally, the absorbance of each well was measured at 492 nm in triplicate.

### 3.7. Protective Effect of Insulin Combined with DOP on Liver Cells

Different groups—normal group (5.6 mM glucose), control group (40 mM glucose) and experimental group (40 mM glucose with 100 μg/mL DOP, 100 μg/mL insulin, 50 μg/mL DOP + 50 μg/mL insulin or 0.625 mg/mL hydrogel, respectively)—were tested. The HL-7702 cells were cultured in a 6-well plate with 2 × 10^5^ cells per well for 24 h, and then different groups of samples were added to the wells and cultured for 24 h. Glucose consumption of HL-7702 cells was measured using a glucose assay kit. Meanwhile, the activity of superoxide dismutase (SOD) and the level of malondialdehyde (MDA) in HL-7702 cells were also detected with an SOD assay kit and MDA assay kit. The reactive oxygen species (ROS) were measured using the DCFH-DA (2’,7’-dichlorofluorescein diacetate) method, which depends on the change in the fluorescence intensity of an active oxygen fluorescent probe indicator, DCFH-DA, to judge the content of active oxygen [51]. After culturing for 24 h with different groups, the stained cells were imaged with a fluorescence microscope.

### 3.8. Statistical Analysis

Statistical analysis was analyzed by one-way ANOVA through SPSS 17.0 software, and all data expressed as mean ± SD. Meanwhile, the significant differences between groups were evaluated by the Tukey post-hoc test, and *p* < 0.05 was considered statistically significant.

## 4. Conclusions

A new type of glucose- and pH-responsive hydrogel based on *Dioscorea opposita* Thunb polysaccharides was first reported in this paper. The formation of a dynamic boric acid bond between the cis-o-diol of DOP and boric acid of PEI-PBA endowed the hydrogel with a dual-sensitive property. Meanwhile, the honeycomb 3D network structure provided the possibility for insulin loading. When glucose competed with DOP to combine with PBA to form a new borate ester bond, insulin was released slowly due to the collapse and destruction of the 3D network structure, and the released insulin could cooperate with bioactive DOP to protect hepatocytes from damage caused by high glucose. In addition, the low toxicity and good biocompatibility also provided a basis for the hydrogel as an intelligent carrier for controlled drug release. It should be noted that the gelation process conditions of the hydrogels are mild, and do not need material modification. In conclusion, the DOP/PEI-PBA hydrogel is expected to be used as an ideal controlled release carrier for insulin for the clinical treatment of diabetes patients with insulin deficiency.

## Figures and Tables

**Table 1:** Mathematic modeling of insulin release.

Release Medium	Model									
	Zero Order		First Order		Ritger–Peppas			Sigmoidal		
	*k*_0_	R^2^	*k*_1_	R^2^	*K*	*n*	R^2^	*k_s_*	*t*_50_	R^2^
Glucose (0 mg/mL)	0.52	0.3875	0.59	0.8608	36.45	0.15	0.9619	0.53	1.48	0.9863
Glucose (1 mg/mL)	0.65	0.4460	0.46	0.8606	36.54	0.18	0.9608	0.54	2.60	0.9866
Glucose (4 mg/mL)	0.79	0.5175	0.38	0.8547	37.13	0.20	0.9713	0.50	4.44	0.9892
pH 7.4	0.57	0.4042	0.48	0.8788	35.46	0.17	0.9567	0.59	1.89	0.9862
pH 5.0	0.64	0.4465	0.48	0.8528	36.84	0.17	0.9667	0.50	2.65	0.9875
pH 2.5	0.68	0.4504	0.53	0.8309	41.03	0.17	0.9739	0.42	3.37	0.9851

## Data Availability

Not applicable.
